# The novel *cis*-encoded antisense RNA AsrC positively regulates the expression of *rpoE*-*rseABC* operon and thus enhances the motility of *Salmonella enterica* serovar typhi

**DOI:** 10.3389/fmicb.2015.00990

**Published:** 2015-09-17

**Authors:** Qi Zhang, Ying Zhang, Xiaolei Zhang, Lifang Zhan, Xin Zhao, Shungao Xu, Xiumei Sheng, Xinxiang Huang

**Affiliations:** ^1^Department of Biochemistry and Molecular Biology, Jiangsu University – School of MedicineZhenjiang, China; ^2^Danyang People’s Hospital of Jiangsu ProvinceDanyang, China

**Keywords:** *Salmonella enterica* serovar typhi, antisense RNA, AsrC, invasion, *rseC*, *rpoE*

## Abstract

Bacterial non-coding RNAs are essential in many cellular processes, including response to environmental stress, and virulence. Deep sequencing analysis of the *Salmonella enterica* serovar typhi (*S. typhi*) transcriptome revealed a novel antisense RNA transcribed in *cis* on the strand complementary to *rseC*, an activator gene of sigma factor RpoE. In this study, expression of this antisense RNA was confirmed in *S. typhi* by Northern hybridization. Rapid amplification of cDNA ends and sequence analysis identified an 893 bp sequence from the antisense RNA coding region that covered all of the *rseC* coding region in the reverse direction of transcription. This sequence of RNA was named as AsrC. After overexpression of AsrC with recombinantant plasmid in *S. typhi*, the bacterial motility was increased obviously. To explore the mechanism of AsrC function, regulation of *rseC* and *rpoE* expression by AsrC was investigated. We found that AsrC increased the levels of *rseC* mRNA and protein. The expression of *rpoE* was also increased in *S. typhi* after overexpression of AsrC, which was dependent on *rseC*. Thus, we propose that AsrC increased RseC level and indirectly activating RpoE which can initiate *fliA* expression and promote the motility of *S. typhi*.

## Introduction

Non-coding RNAs (ncRNAs) are important in gene expression and regulation. In general, their transcription is activated in response to specific growth and stress conditions and their activities aid cells in recovering from stress (Waters and Storz, 2009). ncRNAs activities in controlling virulence and pathogenesis have been demonstrated for a number of gram-negative and gram-positive bacteria (Romby et al., 2006; Toledo-Arana et al., 2007). Most ncRNAs act by forming base pairs with target mRNAs. These base-pairing ncRNAs fall into two categories: *trans*-encoded ncRNAs and *cis*-encoded ncRNAs. The most extensively studied *trans*-encoded RNAs are coded by DNA at genomic locations distant from the mRNAs they regulate. They are thus only partially complementary to their target RNA(s) and regulate mRNAs by short, imperfect base-pairing interaction. *Cis*-encoded RNAs are transcribed from the DNA strand opposite the gene on bacterial chromosomes; these RNA are called antisense RNAs (asRNAs), and thus have perfect complementarity with their target (Storz et al., 2011).

*Cis*-encoded asRNAs are involved in a number of cellular processes in bacteria including replication initiation, conjugation efficiency, suicide, transposition, mRNA degradation, and translation initiation (Thomason and Storz, 2010). The known functional mechanisms employed by asRNAs include transcription attenuation, translation inhibition, promotion, or inhibition of mRNA degradation and preventing the formation of an activator RNA pseudoknot (Georg and Hess, 2011). Transcription of asRNAs has been discovered by computational prediction, oligonucleotide microarrays, or deep sequencing (Thomason and Storz, 2010). By deep RNA sequencing analysis of the transcriptome, we recently found many novel asRNAs in *Salmonella enterica* serovar typhi (*S. typhi*). Some have been found to be important in bacterial replication (Dadzie et al., 2013, 2014). One of the asRNAs coded from the strand opposite the *rseC* gene gets our attention.

The *rseC* gene is fourth in a four-gene operon (*rpoE*, *rseA*, *rseB*, *rseC*) and is cotranscribed with *rpoE*, *rseA*, and *rseB* in *S. typhi*. The regulatory protein σ^E^ (encoded by *rpoE*) is a member of the extracytoplasmic family of alternative sigma factors (Dartigalongue et al., 2001). The protein σ^E^ guides the core RNA polymerase in binding to the promoter region to initiate transcription of specific genes in response to the disturbance of envelope homeostasis caused by the accumulation of unfolded outer membrane proteins, damage to the outer membrane or oxidative and osmotic stress (Mecsas et al., 1993; Missiakas and Raina, 1997; Miticka et al., 2003). In unstressed conditions, σ^E^ activity is negatively regulated by RseA, an inner membrane protein that sequesters σ^E^ in an inactive form at the inner membrane (De Las Penas et al., 1997). Binding of RseB to the periplasmic domain of RseA might shift RseA to a conformation in which it is most effective as an anti-sigma factor, thus keeping σ^E^ activity low (De Las Penas et al., 1997). In contrast, RseC positively modulates the transcriptional activity of σ^E^ (Missiakas et al., 1997).

We describe here the identification and characterization of a novel *cis*-encoded antisense RNA of *S. typhi rseC*, which was named antisense RNA of *rseC* (AsrC), and demonstrate that the expression of this antisense RNA is critical for the motility of *S. typhi*.

## Materials and Methods

### Bacterial Strains, Plasmids, and Growth Conditions

Strains and plasmids used in this study are in **Table 1**. *S. typhi* GIFU10007, a z66-positive wild-type strain was used as the parent strain for all mutants generated in this study. Unless otherwise noted, bacteria were incubated at 37°C in Luria-Bertani (LB) medium with shaking (250 rpm). When necessary, L-arabinose was added to induce the expression of plasmid-borne genes and antibiotics added to cultures were ampicillin (Ap; 100 μg/ml) and kanamycin (50 μg/ml).

**Table 1:** Bacterial strains and plasmids used in this study.

	Relevant characteristics	Source
**Strains**		
*Salmonella typhi* GIFU 10007	Wild type strain; z66^+^	Gifu University Kohbata et al., 1986
ΔP*_*asrC*_*	AsrC promoter mutant stain; Km^r^	This study
WT+pBAD	GIFU 10007 contained pBAD empty vector	This study
WT+pBAD-*asrC*	GIFU 10007 contained pBAD-*asrC*	This study
Δ*rseC*	*rseC* deletion mutant of GIFU 10007	This study
Δ*rseC*+pBAD	Δ*rseC* contained pBAD empty vector	This study
Δ*rseC*+ pBAD-*asrC*	Δ*rseC* contained pBAD-*asrC*	This study
*E. coli* DH 5α	*E. coli* host strain of T vector	Laboratory stock
**Plasmids**		
pBAD/*Myc*-His A	P_lacO_ promoter; Amp^r^	Invitrogen
pBAD-*asrC*	P_lacO_ promoter, as-RseC insert; Amp^r^	This study
pGMB151	Suicide plasmid; *sacB*; Amp^r^	Huang et al., 2004
pET-28a-c(+)	Km^r^	Laboratory collection
pKD46	Temperature-sensitive lambda red recombinase expression plasmid; Amp^r^	Laboratory collection
pGEM-T vector	TA clone; Amp^r^	Promega

### Strain Construction

RseC mutant (Δ*rseC*), with whole ORF deleted, was conducted using a λ Red disruption system according to the method described previously with some modifications (Datsenko and Wanner, 2000). Briefly, a kanamycin-resistant gene was amplified by overlap-extension PCR from the template plasmid pET-28a with primers *rse*C-K1F/*rse*C-K1R and *rse*C-K2F/*rse*C-K2R, which apart from the region of complementarity with the *kan* sequences they were flanked with 89 bp ends homologous for the *rse*C gene. Electrocompetent cells containing the helper plasmid pKD46 were transformed with the purified PCR product. Successful recombinants were recovered by antibiotic selection and screened by PCR with the primers *rse*C-K2F and *rse*C-K2R for correct gene disruption. The promoter region of *asrC* (from +19 to -481) was deleted as described above using primer pairs AsrC-KF and AsrC-KR. Kanamycin-resistant gene was flanked by 50 bp of *asrC* sequences. *AsrC*-promoter mutant stain (ΔP*_asrC_*) was verified by PCR with primers AsrC-KF and AsrC-KR. *S. typhi* wild-type strain derivatives with chromosomally encoded FLAG-fusion proteins were constructed using the sucrose-sensitive suicide vector pGMB151. All constructs were verified by PCR and DNA sequencing.

### Plasmid Construction

To construct the recombinant vector pBAD-*asrC*, a 1034 bp *Nco* I*-Hind* III fragment corresponding to the 893 bp *asrC* sequence and 140 bp upstream of *asrC* was amplified by PCR with primers pBAD-*asrC* PA/PB. Genomic DNA of the wild-type strain GIFU10007 was used as template. The amplicon was cloned into *Nco* I-*Hind* III sites of the vector pBAD/*Myc*-His A (Invitrogen). The wild-type strain GIFU10007 was transformed with the recombinant vector and used as the AsrC-overexpressing strain.

To construct a C-terminal 3 × FLAG-tagged fusion protein, primers *rseC*-F1A/1B, and *rseC-*F2A/2B were used to amplify fragments F1 (422 bp) and F2 (508 bp) from upstream and downstream of the *rseC* gene, respectively, using chromosomal DNA and overlap-extension PCR with *Pfu* Ultra DNA polymerase. FLAG sequences were integrated at the end of the *rseC* gene. F1 and F2 were used as template for a second PCR using primers *rseC*-F1A/F2B to obtain a 900 bp fragment with 66 bp FLAG DNA sequences. The 900 bp fragment was inserted into the *Bam* HI site of the suicide plasmid pGMB151, which carries a sucrose-sensitivity *sacB* gene. The suicide plasmid with the insert was electroporated into the *S. typhi* wild-type strain. Epitope insertion mutant strains cultured on LB plates with sucrose were screened by PCR with primers *rseC*-F1A/F2B.

### RNA Extraction

Overnight cultures of *S. typhi* were diluted 1:100 in LB medium and grown at 37°C with shaking. To determine expression at different times, samples were taken at OD_600_ 0.3, 0.8, 1.2, and 1.8. To determine expression under different stress conditions, bacteria were grown to OD_600_ 0.4 and treated with 0.3 M NaCl for high osmotic stress or 4 mM hydrogen peroxide for oxidative stress. For acid stress, the pH of the culture medium was adjusted with HCl to 4.5. Cells were grown for 30 min after stress. For overexpression analysis, overnight cultures of bacteria carrying an empty pBAD/*Myc*-His A plasmid (WT+pBAD) and a plasmid expressing AsrC (WT+pBAD-*asrC*) were diluted 1:100 in LB medium and grown at 37°C to OD_600_ 0.4. Overexpression was induced by addition of 0.2% (w/v) L-arabinose. Aliquots were taken prior to addition or maintained for the indicated time after L-arabinose addition. To extract total RNA, cultures were pelleted by centrifugation at 10,000 *g* for 2 min and RNAs were isolated using TRIzol (Invitrogen). RNA samples were treated with DNase I (Takara) to eliminate DNA contamination and quantified by ND-1000 Spectrophotometer (NanoDrop Technologies, Wilmington, DE, USA).

### 5′ and 3′ RACE Analysis

5′-rapid amplification of cDNA ends (RACEs) used 5′-Full RACE kits (Takara) according to the manufacturer’s instructions. Briefly, 5 μg of total RNA was treated with 10 units of calf intestine alkaline phosphatase (1 h, 50°C) and 1 unit of tobacco acid pyrophosphotase (1 h, 37°C) and ligated to 250 pmol of supplied 5′ RACE adaptor with 40 unit of T4 RNA ligase (1 h, 16°C). Reverse transcription was at 42°C for 1 h with 5 units M-MLV reverse transcriptase and 25 pmol antisense RNA specific primer (5′ RACE RT). The cDNA was amplified with an adaptor-specific primer (5′ RACE outer primer) and *asrC* specific primer (5′ RACE GSP1). A second amplification was with 5′ RACE inner primer and 5′ RACE GSP2 using first PCR products as template. Purified PCR products were cloned into pGEM-T vector (Promega). Bacterial colonies were checked for appropriate inserts by PCR and confirmed by sequencing. The protocol for 3′-RACE was as described previously (Argaman et al., 2001). Briefly, total RNA (15 μg) was dephosphorylated with calf intestine alkaline phosphatase (Takara). RNA was ligated to 5′-phosphorylated 3′-RACE adaptor. Reverse transcription was performed as described for 5′-RACE with 3′-RACE adaptor-specific primer. Nested PCR was performed with 3′-RACE adaptor primer and *asrC*-specific primers (3′-RACE GSP1 and 3′-RACE GSP2). Cloning and sequence analysis were as described above.

### Northern Hybridization Analysis

Total RNA (5–30 μg) was separated on 7 M urea/6% polyacrylamide gels in 0.5 × TBE buffer and transferred to Hybond N^+^ membranes (GE Healthcare). Oligonucleotides (**Table 2**) were 5′-labeled with [γ-^32^P]-ATP by T4 polynucleotide kinase (Takara). Following prehybridization of membranes in Hyb hybridization buffer (Innogent) for 1 h at 42°C, membranes were hybridized overnight at 42°C. Membranes were washed twice with 2 × SSC + 0.05% SDS and twice with 0.1 × SSC + 0.1% SDS and exposed to KODAK X-Ray film at -70°C.

**Table 2:** Primers and oligonucleotides used in this study.

Name	Sequence (5′ to 3′)
**Primers used to construct strains**	
pBAD-*asrC* PA	ATCCATGGTAACGAACGGGAAACTTC
pBAD-*asrC* PB	GCAAGCTTATCAAATTCAGGGCAGTA
*rseC*-F1A	ATAGGATCCCGCAAACCACGCATACCATT
*rseC*-F1B1	TTTATAATCACCGTCATGGTCTTTGTAGTCCTGGCGCGTTTCGATTGAGG
*rseC*-F1B2	TTATTTATCGTCGTCATCTTTGTAGTCGATATCATGATCTTTATAATCACCGTCATGGT
*rseC-* F2A	ATCGACTACAAAGATGACGACGATAAATAAACGTTTTGCCCAATGGTACA
*rseC*-F2B	ATAGGATCCAAGTTTCACCATCAGACGCT
*rseC*-FA	GCTATTCCCGAAAACTGGCG
*rseC*-FB	CTGTAAAACACCGCAACGCA
AsrC-KF	ATCGACGCTGTCTGACCGTATTATCCAAATCTGCGGTGGCCTGTCTGACCGGTCTGACGCTCAGTGGA
AsrC-KR	ATGGTGCCGTTTTTAATACGCACCAGGGATACCACGCCCAGGTAGTTATCTTTCGGCCTATTGGTTAA
*rseC*-K1F	CCGCAAACGGCGAAGCGCATTGCCGACAGTATCAAATTCAGGGCAGTACAGGTCTGACGCTCAGTGGA
*rseC*-K1R	GGACAACAACCCAACGCGGCAGGTGTACCATGTACCATTGGGCAAAACGTTTTCGGCCTATTGGTTAA
*rseC*-K2F	CGCGACAACGCGGAAATCACCATTGTCGGAGAGCTTCCGCCGCAAACGGCGAAGCGCAT
*rseC*-K2R	TGTAAAACACCGCAACGCAGGAAAAGCGCATGCTTATCGGGACAACAACCCAACGCGGC
**Primers and adaptor used for 5′ and 3′ RACE**	
5**′** RACE RT	CAGGGCAGTACAATGATTAAAGAG
5**′** RACE GSP1	GATTAAAGAGTGGGCAACCGT
5**′** RACE GSP2	TATCTCCTGGCAGAATGGTC
5**′** RACE outer primer	CATGGCTACATGCTGACAGCCTA
5**′** RACE inner primer	CGCGGATCCACAGCCTACTGATGATCAGTCGATG
3**′** RACE GSP1	AGGCGACATATAGACCAG
3**′** RACE GSP2	TTTATTCAGCACGCGACT
3**′** RACE adaptor	5**′**-phosphate-UUCACUGUUCUUAGCGGCCGCAUGCUC-idT-3**′**
3**′** RACE adaptor primer	GGCCGCTAAGAACAGTGAA
**Primers used for Real-time PCR analyses**	
*asrC*-qF	AGGCGACATATAGACCAG
*asrC*-qR	TCAAATTCAGGGCAGTAC
*rseC*-qF	TCAAATTCAGGGCAGTAC
*rseC*-qR	GCTGCATGACGCTTTAAC
*rpoE*-qF	ACCAGCATAAAGTGGCGAGT
*rpoE*-qR	TACATCACTGGAAGGCGGAC
*flhD*-qF	AGCGTTTGATCGTCCAG
*flhD*-qR	CGTCCACTTCATTGAGCA
*fliA*-qF	GTGGAACTGGACGATCTGCT
*fliA*-qR	TCTGACGATACTCCGCAACA
*fliD*-qF	TTCGCAACCACCAAAGAGCA
*fliD*-qR	GCGGTAAGCACCAACTGGAA
5S-qF	TTGTCTGGCGGCAGTAGC
5S-qR	TTTGATGCCTGGCAGTTC
**Probes used for Northern hybridization**	
AsrC-P1	ATTCAGGGCAGTACAATGATTAAAGAGTGGGCAACCGTTATCTCCTGGCAGAATG
AsrC-P2	GCCTGGCAGCCCGTGATTTTAAACGTTGCCCTCCCGCCTGACCTTGTCCGCGTCGAAAC
*rseC*-P	GTTTCGACGCGGACAAGGTCAGGCGGGAGGGCAACGTTTAAAATCACGGGCTGCCAGGC
*rpoE*-P	AATGATTCCTGTACGACATCGGGAACGTCGCCCGATGGCACATAGCGGGAAACCAGACT
5S-P	CTACCATCGGCGCTACGGCGTTTCACTTCTGAGTTCGGCATGGGGTCAGGTGGGA

### Quantitative Real-Time PCR

DNase I-treated total RNA (4 μg) was used for cDNA synthesis with PrimeScript Reverse Transcriptase (Takara) and gene-specific primers according to the manufacturer’s protocol. Quantification of cDNA used SYBR *Premix Ex Taq* II (Takara) and appropriate primers and was monitored using a C1000 Thermal Cycler (Bio-Rad) according to manufacturer’s instructions. Primer sequences are in **Table 2**. All samples were normalized against levels of 5S ribosomal RNA amplified with primers 5S-qF and 5S-qR. Relative mRNA levels were determined by the comparative CT method (Livak and Schmittgen, 2001). Each experiment was performed in triplicate.

### Motility Assay

Swimming motility was evaluated on LB plates containing 0.3% (w/v) agar. Bacterial strains were grown in LB broth at 37°C overnight, diluted 1:100 and incubated to OD_600_ 0.4. L-arabinose was added to induce AsrC expression for 1 h. Bacterial cultures (2.5 μl) were incubated on spots on swimming plates at 37°C for 8 h. Motility was assessed by measuring the circular swim formed by the growing motile cells. Experiments were carried out triplicate.

### Western Blot Analysis

After L-arabinose-induction, bacterial cells were cultured for the indicated time (normalized to the optical density at OD_600_) before harvest, pelleted, and washed once with an equal volume of PBS buffer. Final pellets were resuspended in PBS buffer and maintained on ice during sonication for the minimum time required to observe cleared lysate (10 s each at 1 min intervals) with an CL4 probe in a XL2020 Ultrasonic Processor (Misonix, Inc., Farmingdale, NY, USA). Supernatants of bacterial cultures after ultrasonic treatment were used for Western blots. Samples were separated on 15% polyacrylamide gels and proteins were electrophoretically transferred to polyvinylidene difluoride membranes. Membranes were treated with 5% non-fat dried milk in Tris-buffered saline, incubated with anti-FLAG (CMC Scientific), anti-RpoE (Santa Cruz Biotechology), or anti-DnaK antibodies (Enzo Life Sciences) raised in mice and subsequently incubated with anti-mouse immunoglobulin G linked to horseradish peroxidase. Crossreactive proteins were detected with ECL plus Western blotting detection reagents (Thermo Scientific).

### Statistical Analysis

Data are presented as means ± SE. Statistical analysis was performed with Student’s *t*-test. Significance was defined as *P <* 0.05.

## Results

### Identification and Characterization of AsrC Expression in *S. typhi*

**FIGURE 1:**
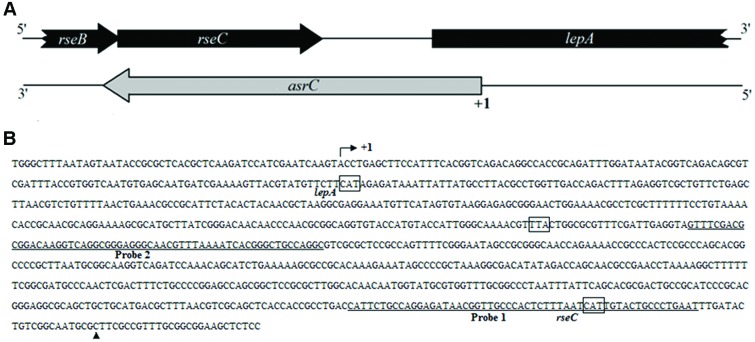
**Schematic representation and sequence analyses of *asrC*. (A)** Genomic location of *asrC*. Black arrows, location of *rseB*, *rseC*, and *lepA* genes; gray arrows, position of *asrC*. **(B)** Sequence analysis by RACE of the AsrC. Bent arrow, transcription start site; +1 and ▲, experimentally determined transcription start site and transcription stop site of the *asrC*, respectively; underlined sequence, two different positions of oligonucleotide probes used for Northern hybridization for AsrC expression.

By transcriptome analysis of *S. typhi*, several new ncRNAs were found, including a putative antisense RNA for *rseC*. To determine the boundaries of the putative novel ncRNA, rapid amplification of cDNA ends (RACEs) was used. Results of 5′-RACE and 3′-RACE revealed the 5′-end of the transcript, which was 119 bp downstream of the *lepA* start codon. The 3′-end of the transcript was 36 bp upstream of the *rseC* start codon, indicating a full-length transcript of 893 nt. The gene structure and sequence of the transcript are in **Figure 1**. End mapping revealed that the novel transcript was transcribed in *cis* from the strand complementary to *rseC* and overlapped the entire *rseC* mRNA. We therefore named it AsrC. From the entire sequence of the novel asRNA, no obvious ORF structure longer than 150 nt or SD element was found. Thus, we hypothesized that the novel asRNA was an ncRNA.

**FIGURE 2:**
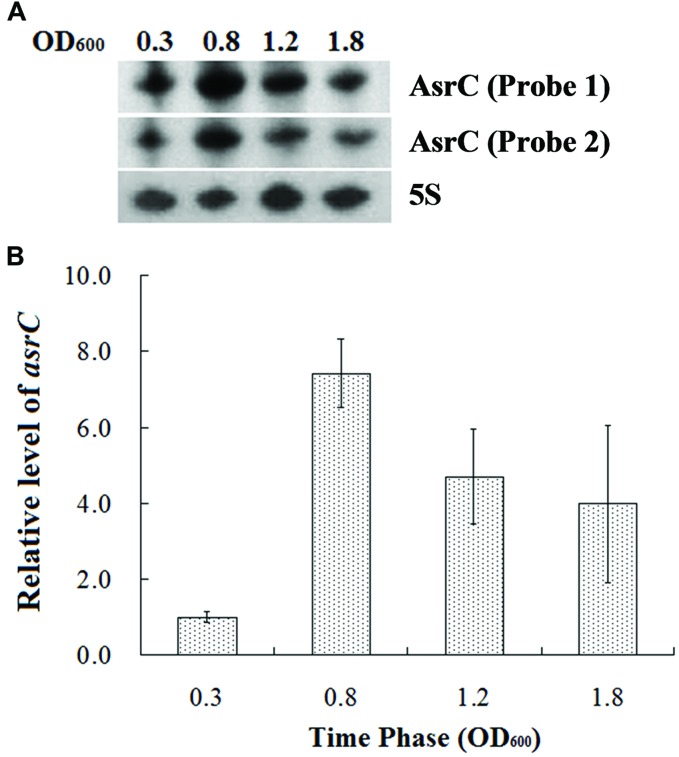
**Expression of AsrC at different times. (A)** Total RNA was extracted at OD_600_ 0.3, 0.8, 1.2, and 1.8 and 5 μg total RNA was loaded into gels, separated on a 7 M urea/6% polyacrylamide gels and transferred to a membranes. Probes 1 and 2 for two regions were used for Northern hybridization. 5S rRNA was the loading control. **(B)** qRT-PCR to measure AsrC at different times. 5S rRNA was the internal reference.

**FIGURE 3:**
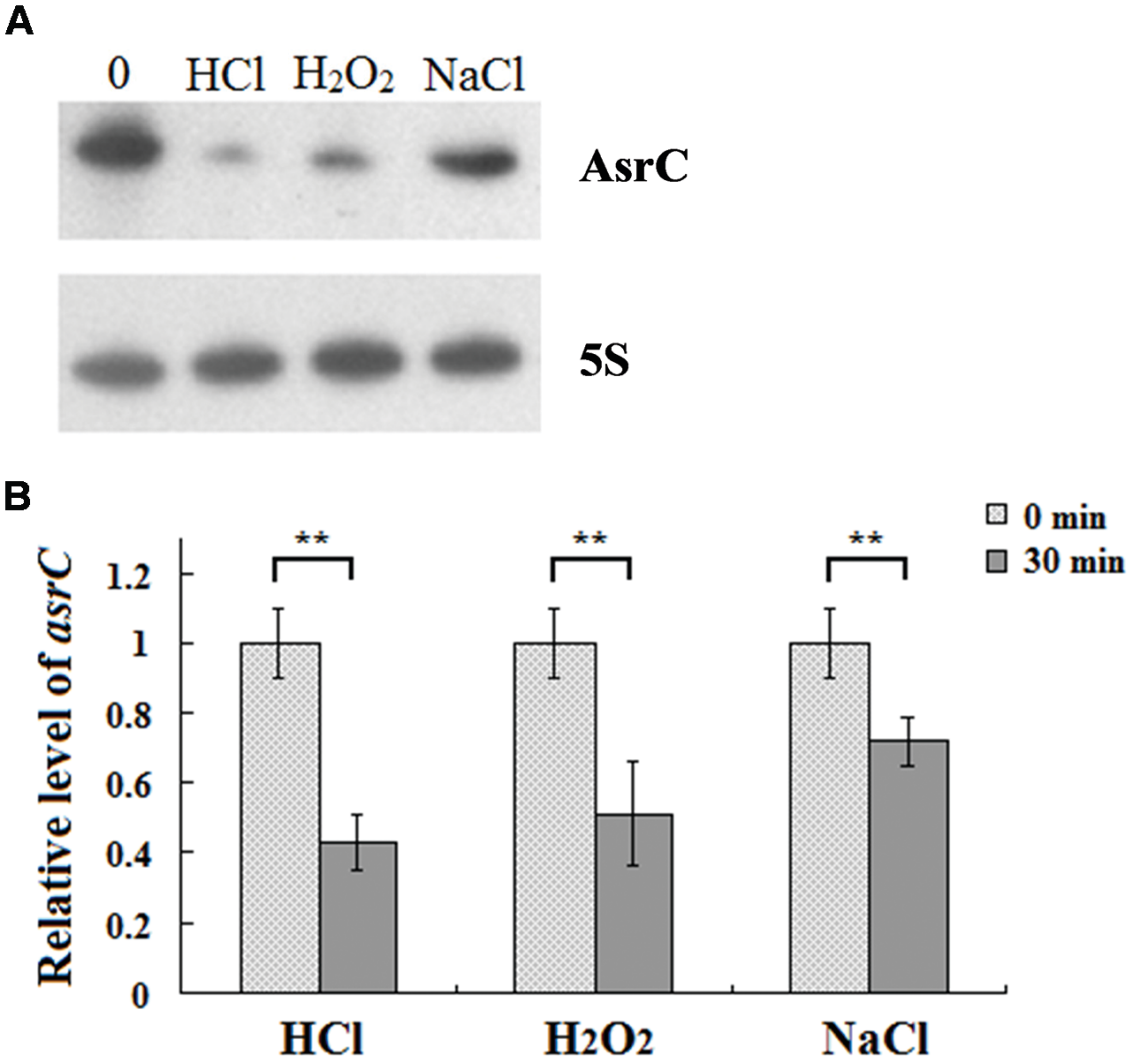
**Expression of AsrC under stress conditions. (A)** Northern hybridization and **(B)** qRT-PCR of total RNA isolated from *Salmonella typhi* cells grown in LB to OD_600_ 0.4 and subjected for 30 min to acid stress (HCl: pH 4.5), oxidative stress (H_2_O_2_: 4 mM hydrogen peroxide) or osmotic shock (NaCl: 0.3 M NaCl). Internal reference was 5S rRNA. ^∗∗^*P* < 0.01 compared with control group.

To understand the expression characteristics of *asrC*, RNA was harvested from wild-type *S. typhi* at different times and under different stress conditions and analyzed by Northern hybridization and qRT-PCR. Total RNA from a *S. typhi* wildtype strain was collected at OD_600_ 0.3, 0.8, 1.2, and 1.8, representing bacterial growth phases from lag through stationary. Two oligonucleotide probes specific to different regions of the *asrC* gene (**Figure 1B**) were designed for Northern hybridization. AsrC RNA was detected by both probes mainly in late log and early stationary phase (**Figure 2A**). The expression pattern of *asrC* was observed by qRT-PCR (**Figure 2B**). Expression of *asrC* was also determined under stress conditions reflecting the *Salmonella* environment upon invasion of a host or within macrophages. Total RNA was extracted from wild-type after cells were subjected to acidic, oxidative, or osmotic stress. Northern hybridization and qRT-PCR analysis showed that expression of *asrC* was reduced in acidic, oxidative conditions (**Figures 3A,B**). These results suggested that the novel asRNA AsrC was expressed in *S. typhi* and that expression was regulated by environmental conditions.

### AsrC Eexpression Enhances *S. typhi* Motility

**FIGURE 4:**
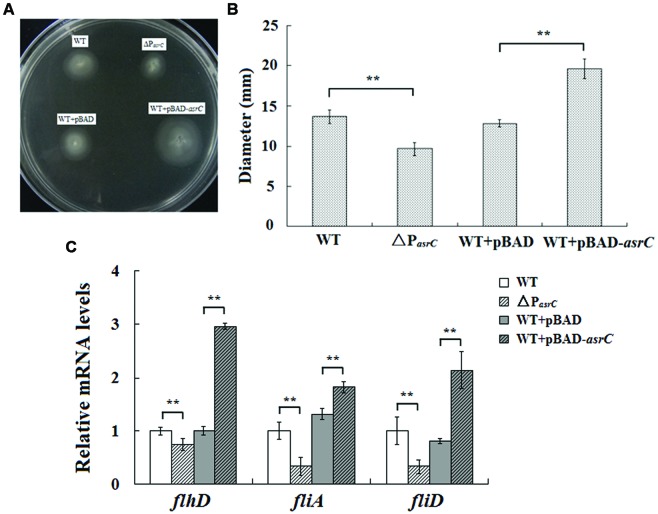
**Motility assay of the wild type, ΔP*_asrC_*, WT+pBAD and WT+pBAD-*asrC* strains. (A)** AsrC-promoter mutant strain with decreased motility. Overexpression of AsrC increased motility on swim-agar plates. **(B)** Motility ring diameters of *S. typhi* strains. Bacterial were spotted onto LB plates with 0.3% agar and incubated at 37°C for 8 h. The image is representative of three independent experiments. **(C)** mRNA of flagellar genes (*flhD*, *fliA*, and *fliD*) determined by qRT-PCR. RNA was extracted from three independent cultures for each strain grown to OD_600_ 0.4. Levels of 5S rRNA were the internal reference. ^∗∗^ *P* < 0.01 compared with control group.

To investigate the function of the AsrC, the *asrC*-promoter mutant stain (ΔP*_asrC_*) of *S. typhi* was prepared. qRT-PCR results showed that *asrC* had almost no expression in ΔP*_asrC_* strain (Supplementary Figure S1). Overexpression of AsrC was performed in *S. typhi* which was transformed by a recombinant plasmid pBAD-*asrC* and induced with L-arabinose. The motility of *S. typhi* influence by the AsrC was assessed by using motility-swim agar plates. The motility of ΔP*_asrC_* was significantly decreased compared to the wild-type strain (WT). After overexpression of AsrC, *S. typhi* (WT+pBAD-*asrC*) had significantly increased motility compared to the control strain (WT+pBAD; **Figures 4A,B**). Transcript levels of flagellar genes *flhD*, *fliA*, and *fliD* were compared among WT, ΔP*_asrC_*, WT+pBAD, and WT+pBAD-*asrC* strains using qRT-PCR. Expression of *flhD*, *fliA*, and *fliD* in the mutant stain ΔP*_asrC_* was decreased compared to the WT strain and increased in the WT+pBAD-*asrC* strain compared to the empty-plasmid control (**Figure 4C**). *LepA* complementary strain and control strain were constructed by transferring the recombinant plasmid pBAD*-lepA* and the empty vector pBAD/gIII into the ΔP*_asrC_* mutant strain. Expression of flagella-related genes of the two strains was observed to be identical (data not shown). These results suggested that AsrC can up-regulate *S. typhi* motility.

### Overexpression of AsrC Increases *rseC* mRNA and Protein Levels

**FIGURE 5:**
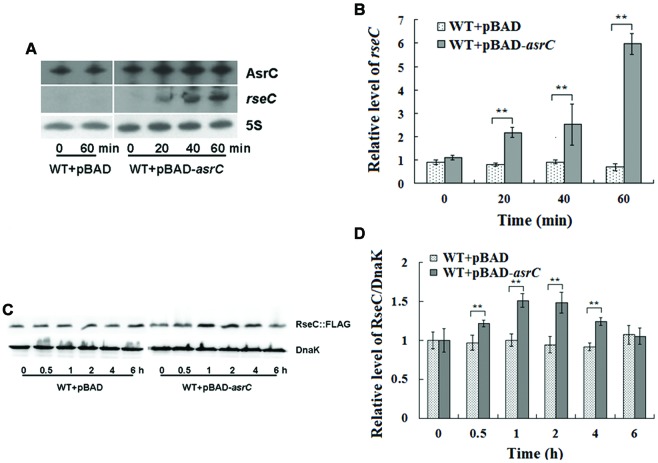
**Overexpression of AsrC increased *rseC* mRNA and protein expression levels.** mRNA levels of *resC* were assessed by Northern hybridization **(A)** and qRT-PCR **(B)**. Total RNA from wild-type (WT+pBAD) and overexpression strain (WT+pBAD-*asrC*) grown to OD_600_ 0.4 at 0, 20, 40, and 60 min after addition of L-arabinose (0.2% final concentration). Levels of 5S rRNA were the internal reference. **(C)** Western blot profile of RseC protein relative to DnaK in WT+pBAD and WT+pBAD-asrC strains. Representative blots are shown. **(D)** Quantitation of RseC protein based on Western blots. Proteins were isolated from WT+pBAD and WT+pBAD-*asrC* strains grown to OD_600_ 0.4 at 0, 0.5, 1, 2, 4, and 6 h after addition of L-arabinose (0.2% final concentration). Experiments were repeated three times and error bars indicate standard deviations. ^∗∗^*P* < 0.01 compared with control group.

AsrC is transcribed from the DNA strand opposite the *rseC* gene and therefore has perfect complementarity with *rseC* mRNA. To investigate the effect of *asrC* expression on *resC* levels, WT+pBAD and WT+pBAD-*asrC* strains were grown to exponential phase (OD_600_ 0.4) and treated with L-arabinose for 0, 20, 40, or 60 min. Changes in mRNA abundance were monitored by Northern hybridization. The mRNA level of *rseC* increased time-dependently when AsrC was overexpressed, but no additional increase was seen in the WT+pBAD strain (**Figure 5A**). These results were confirmed by qRT-PCR. The mRNA level of *rseC* was significantly higher when AsrC was overexpressed compared to the WT+pBAD strain (**Figure 5B**). The effect of AsrC overexpression on the protein level of RseC was analyzed. Western blots showed an increase in RseC protein levels in the WT+pBAD-*asrC* strain compared to the WT+pBAD strain during induction of *asrC* expression for the first 4 h with recovery to basal level at 6 h. No significant expression difference was observed in the WT+pBAD control strain (**Figures 5C,D**). The mRNA level of *rseC* was also detected in ΔP*_asrC_ lepA* complementary (ΔP*_asrC_*+pBAD-*lepA*) strain and WT+pBAD control strain. qRT-PCR results showed that *rseC* expression significantly decreased in ΔP*_asrC_*+pBAD-*lepA* strain compared to WT+pBAD control strain (Supplementary Figure S2). These observations suggested that AsrC might be vital for in increasing *rseC* mRNA and protein levels.

### Overexpression of AsrC Increases *rpoE* mRNA and Protein Levels

**FIGURE 6:**
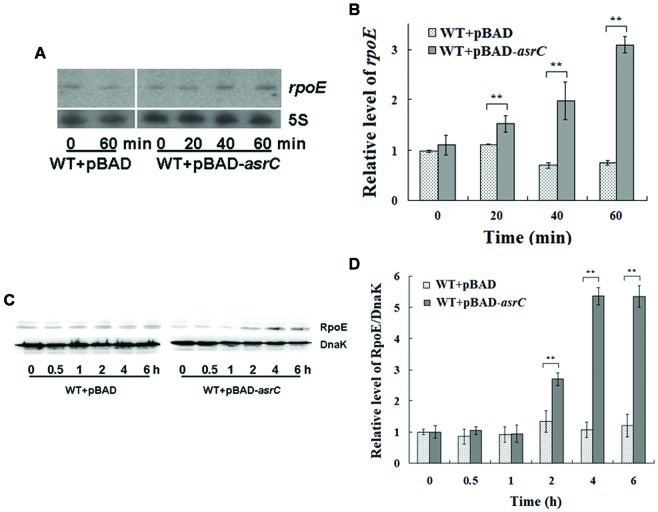
**Overexpression of AsrC increased *rpoE* mRNA and protein.** *RpoE* mRNA assessed by Northern hybridization **(A)** and qRT-PCR **(B)**. Total RNAs were isolated from WT+pBAD and WT+pBAD-*asrC* strains grown to OD_600_ 0.4 at 0, 20, 40, and 60 min after addition of L-arabinose (0.2% final concentration). Levels of 5S rRNA were the internal reference. **(C)** Western blot of RpoE protein relative to DnaK in WT+pBAD and WT+pBAD-*asrC* strains. Representative blots are shown. **(D)** Quantitation of RpoE protein levels based on Western blots. Experiments were repeated three times and error bars indicate standard deviations. ^∗∗^*P* < 0.01 compared with control group.

The last gene of the *rpoE* operon is *rseC*. RseC positively modulates the transcriptional activity of σ^E^ (Missiakas et al., 1997). Thus, we investigated if AsrC overexpression influenced the expression of *rpoE.* Northern hybridization and qRT-PCR both showed that overexpression of AsrC increased *rpoE* mRNA, but the WT+pBAD control strain displayed no significant differences in *rpoE* mRNA level after L-arabinose induction for 60 min (**Figures 6A,B**). The level of *rpoE* mRNA increased about threefold after 60 min of L-arabinose induction in the WT+pBAD-*asrC* strain (**Figure 6B**). RpoE protein increased time-dependently after AsrC overexpression was induced (**Figure 6C**). Western blot quantification showed that RpoE protein was significantly elevated after L-arabinose induction for 2 h and reached a maximum (5.3-fold) after induction for 4 h (**Figure 6D**). Expression of *rpoE* was also detected in ΔP*_asrC_*+pBAD-*lepA* strain and WT+pBAD control strain. qRT-PCR results showed that mRNA level of *rpoE* significantly decreased in ΔP*_asrC_*+pBAD-*lepA* strain compared to WT+pBAD control strain (Supplementary Figure S2). These results indicated that expression of RpoE was positively affected by AsrC.

### AsrC Increases *rpoE* mRNA and Protein Through *rseC*

**FIGURE 7:**
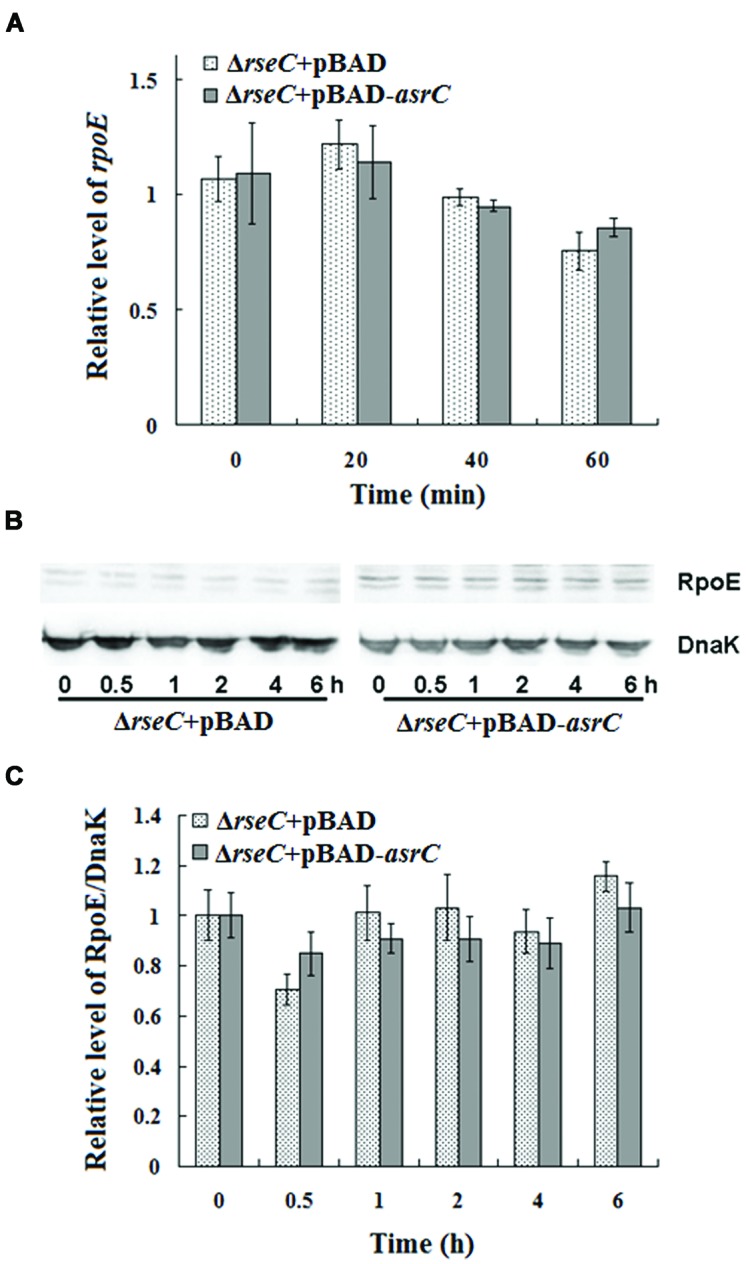
**Expression of *rpoE* mRNA and protein in *rseC* mutant strain after overexpression of AsrC. (A)** qRT-PCR for *rpoE* mRNA levels in Δ*rseC*+pBAD and Δ*rseC*+pBAD-*asrC* strains. Total RNA was isolated from Δ*rseC*+pBAD and Δ*rseC*+pBAD-*asrC* strains grown to OD_600_ 0.4 at 0 min, 20 min, 40 min, and 60 min after addition of L-arabinose (0.2% final concentration). Levels of 5S rRNA were the internal reference. **(B)** Western blot of RpoE protein relative to DnaK in Δ*rseC*+pBAD and Δ*rseC*+pBAD-*asrC* strains. **(C)** Quantitation of RpoE protein based on Western blots. Proteins were isolated from Δ*rseC*+pBAD and Δ*rseC*+pBAD-*asrC* strains grown to OD_600_ 0.4 at 0 h, 0.5 h, 1 h, 2 h, 4 h and 6 h after addition of L-arabinose (0.2% final concentration). Experiments were repeated three times and error bars indicate standard deviations.

The results above indicated that AsrC overexpression increased *rseC* and *rpoE* mRNA and protein. RseC positively modulate σ^E^ (RpoE) activity (Missiakas et al., 1997), suggesting that AsrC might elevate *rpoE* mRNA and protein through increased transcription and translation of *rseC*. We investigated *rpoE* mRNA and protein in an *rseC* mutant containing a pBAD-*asrC* plasmid (Δ*rseC*+pBAD-*asrC*) or pBAD control plasmid (Δ*rseC*+pBAD). No significant expression difference in mRNA or protein was observed between the Δ*rseC*+pBAD-*asrC* strain and the Δ*rseC*+pBAD control strain (**Figures 7A–C**). These results indicated that increased *rpoE* mRNA and protein induced by AsrC overexpression was *rseC* dependent, primarily through increasing *rseC* transcription and translation.

## Discussion

Most ncRNAs are transcribed and accumulate in the stationary phase of bacterial growth and under specific stress conditions (Wassarman et al., 2001; Vogel et al., 2003). Bacteria prepare for these conditions by upregulating a number of genes and regulators to cope with environmental changes. Numerous ncRNAs of bacterial pathogens affect the expression of virulence genes (Pichon and Felden, 2005; Papenfort and Vogel, 2010). RNA III of *Staphylococcus aureus* was the first regulatory ncRNA shown to be involved in bacterial pathogenicity (Novick et al., 1993). Recently, we found many novel asRNAs in *S. typhi* by RNA-seq analysis (Dadzie et al., 2013, 2014). A transcript that overlaps the entire mRNA sequence of *rseC* was found in *S. typhi*. We identified an 893 nt sequence of a putative novel ncRNA by RACE. This sequence was transcribed from 119 nt downstream of the *lepA* start codon to the last 40 nt of *rseB* (**Figure 1**). No obvious ORF structure longer than 150 nt or an SD element was found in the entire sequence. Therefore, we believe this is a novel ncRNA. The novel ncRNA AsrC is transcribed in *cis* from the strand complementary to *rseC* and overlaps the entire *rseC* mRNA.

Using Northern hybridization, we confirmed expression of AsrC in *S. typhi*. We determined the expression characteristics of AsrC in *S. typhi* during different growth phases and under selected stress conditions using Northern hybridization and qRT-PCR.

The abundance of the *asrC* transcript is highest during late exponential phase (**Figure 2**). Transcription of *asrC* was regulated in different environmental conditions (**Figure 3**). Transcription of ncRNAs in general is activated in response to specific growth and stress conditions and their activities aid cells in recovery from those stresses (Waters and Storz, 2009).

According to the expressional characteristics of AsrC, we suspected that is a functional product in *S. typhi*. Effect of AsrC expression on the bacterial phenomenon was investigated at first. In this study, we could not find obvious difference in growth curve between the AsrC mutant strain ΔP*_asrC_* and the wild type strian of *S. typhi* (data not shown). Results obtained from motility assays indicate that expression changes of asrC resulted in variation in swimming motility (**Figure 4**), implying that AsrC plays an important role in bacterial motility. Flagella are cell surface appendages involved in a number of bacterial behaviors, including motility (Chilcott and Hughes, 2000; Yang et al., 2012). The flagellar genes are organized into a transcriptional hierarchy with three promoter classes (class 1, 2, and 3). Class 1 promoters drive expression of the flagellar master operon that encodes the transcription factors FlhD and FlhC. Class 3 flagellar genes are regulated by FliA, encoded by class 2 gene *fliA*, which activates a third group of genes, class 3, needed for final flagellar assembly (Chilcott and Hughes, 2000). The *fliD* gene, which belongs to class 2, encodes the filament-cap protein of the flagellar apparatus (Ohnishi et al., 1990; Ikebe et al., 1999; Chilcott and Hughes, 2000). We used qRT-PCR approach to identify relative mRNA expression of flagella genes *flhD*, *fliA*, and *fliD* in the AsrC mutant and overexpression strains. AsrC mutant decreased the expression of *flhD*, *fliA*, and *fliD* and overexpression of AsrC increased the *flhD*, *fliA*, and *fliD* expression (**Figure 4**). The 5′-end of AsrC is 119 nt downstream of the *lepA* start codon, so mutation of the AsrC promoter region might influence *lepA* expression. Therefore, we constructed a *lepA* complementary strain and control strain by transferring the recombinant plasmid pBAD*-lepA* and the empty vector pBAD/gIII into the ΔP*_asrC_* mutant strain. No significant differences in expression of flagella-related genes were observed compared to pBAD*-lepA* and pBAD control strains (data not shown).

We previously found that RpoE can promote *Salmonella* flagellar gene expression and motility (Du et al., 2011). RseC was reported as a positive modulater of RpoE (σ^E^), as overproduction from a multicopy plasmid led to an increase in transcriptional σ^E^ activity (Missiakas et al., 1997). RseC fine-tunes the interaction of RseA and RseB with σ^E^ and is associated with other bacterial functions. RseC maintains the reduced state of SoxR, one of two transcription factors that sense the presence of oxidants and induce genes against oxidative stress (Koo et al., 2003). RseC is reported to be involved in synthesis of the pyrimidine ring of thiamine, which is a requirement for conversion of aminoimidazole ribotide to the 4-amino-5-hydroxymethyl-2-methyl pyrimidine in *S. enterica* serovar *typhimurium* (Beck et al., 1997). RseC and proteins such as ApbC and ApbE and glutathione participate in Fe-S cluster repair (Skovran et al., 2004). We observed that overexpression of AsrC led to a significant increase in *rseC* mRNA and enhanced its translation, which was confirmed by Western blot analysis (**Figure 5**). This result was consistent with the observation that many sense-antisense pairs exhibit positively coregulated expression profiles that indicate possible involvement of antisense RNAs in stabilizing *cis*-encoded mRNAs (Chinni et al., 2010).

**FIGURE 8:**
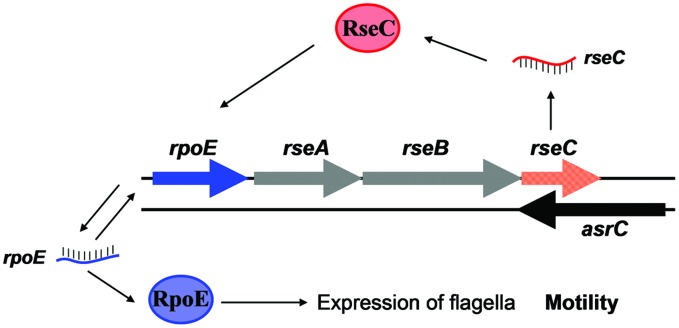
**AsrC and its regulatory circuits.** AsrC expression increases *rseC* mRNA and protein levels. RseC positively modulates transcriptional activity of *rpoE* and increases RpoE (σ^E^) protein. The *rpoE* gene is positively autoregulated. Enhanced σ^E^ regulatory pathways respond by inducing transcription of genes related to motility. Arrows, activation.

The protein σ^E^ is involved in the response to extracytoplasmic stresses and initiates transcription of a series of genes in *Escherichia* and *Salmonella* under environmental stress, for example, high osmolarity and oxidative stress (Testerman et al., 2002; Miticka et al., 2003; Rolhion et al., 2007). In our study, AsrC overexpression increased *rpoE* mRNA and protein, probably by enhancing transcription and translation of *rseC* (**Figure 6**). RseC protein began to increase after 0.5 h of AsrC induction and RpoE increased after 2 h of induction, 1.5 h later than RseC. Increased RpoE protein appeared later than RseC protein, suggesting that AsrC induced RpoE expression through enhancing expression of RseC. Our results showed that AsrC was expressed throughout *S. typhi* growth, with the highest expression during late exponential phase (**Figure 2**), and *rpoE* mRNA were increased ∼16-fold during the transition to stationary phase (Testerman et al., 2002). The enhancement of *rpoE* expression could be partly due to increased expression of *asrC* mRNA, which is resulted in an increase in *rseC* expression. In our observation, overexpression of AsrC in an *rseC* mutant strain had no significant effect on *rpoE* mRNA and protein (**Figure 7**). Thus, we hypothesized a pathway in which AsrC overexpression increased *rpoE* expression primarily through increasing expression of *rseC* and enhanced σ^E^ regulatory pathways. Moreover, it is known that at the transcriptional level, the *rpoE* gene is positively autoregulated, because one of its own promoters is transcribed by the Eσ^E^ holoenzyme itself (Raina et al., 1995; Rouvière et al., 1995). Therefore, by increasing the expression of RseC that positively controls RpoE activity, AsrC could induces the expression of the *rpoE*-*rseABC* operon. Taken together, AsrC promoted the expression of genes related to motility and enhancing the motility of *S. typhi* (**Figure 8**).

In summary, the full-length AsrC was identified to be 893 nt, located 119 nt downstream of the *lepA* start codon and 36 nt upstream of *rseC* start codon. Expression of this antisense RNA increased the levels of *rseC* mRNA and protein and positively regulating RpoE. These processes might be important in motility of *S. typhi*.

## Author Contributions

Conceived and designed the experiments: QZ, YZ, XZ; Performed the experiments: QZ, YZ, XZ, LZ; Analyzed the data: QZ, YZ, XH, SX, XS; Contributed reagents/materials/analysis tools: SX, XZ; Wrote the paper: YZ, XZ, XH, XS.

## Conflict of Interest Statement

The authors declare that the research was conducted in the absence of any commercial or financial relationships that could be construed as a potential conflict of interest.
