# Supplementary material for: The novel cis-encoded antisense RNA AsrC positively regulates the expression of rpoE-rseABC operon and thus enhances the motility of Salmonella enterica serovar typhi
Source: Front Microbiol. 2015 Sep 17;6:990. doi: 10.3389/fmicb.2015.00990 (PMC4585123; doi:10.3389/fmicb.2015.00990)
Supplement: Supplementary file 1 [file Data_Sheet_1.DOC]

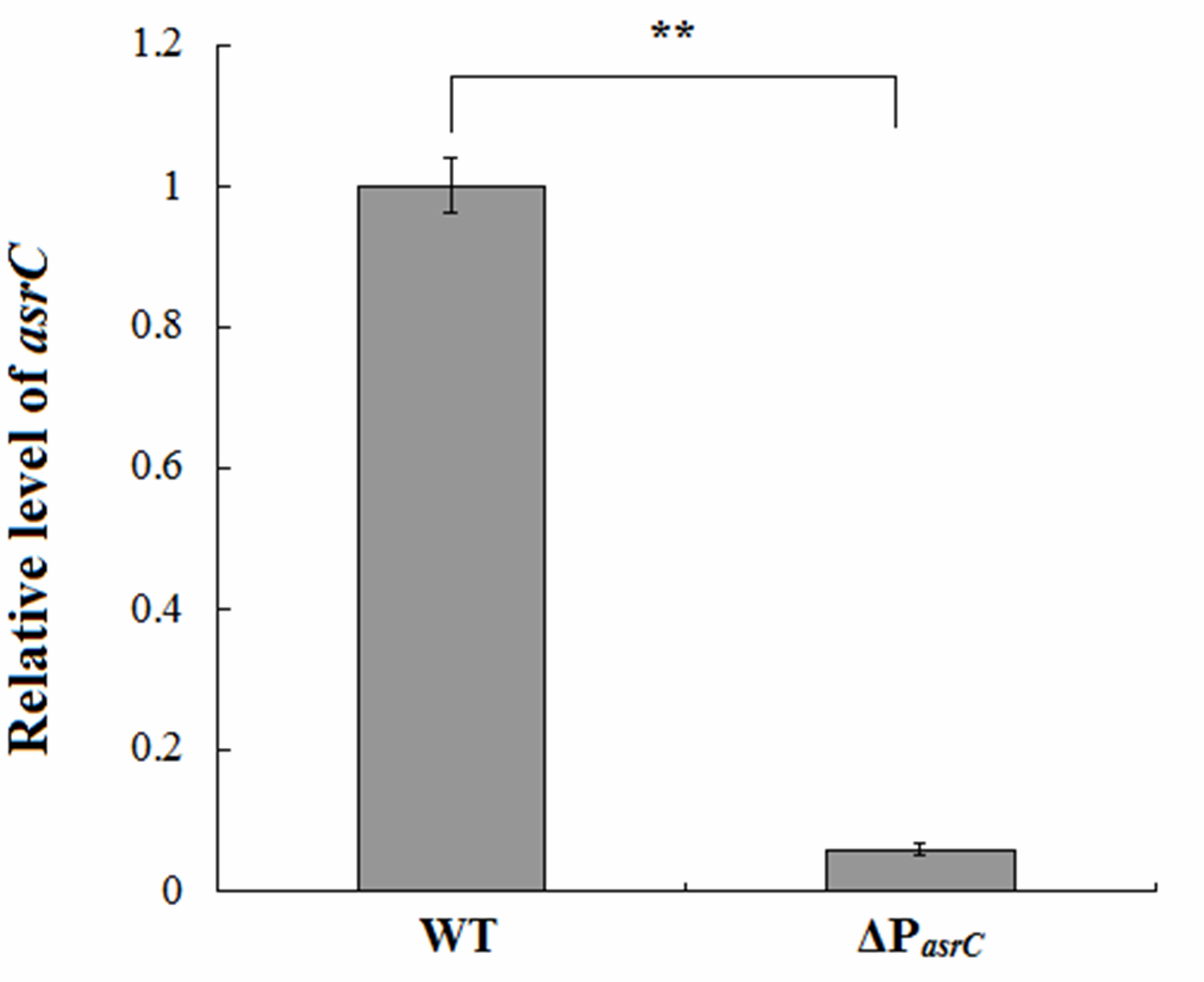


**Figure S1. Expression of AsrC in WT and** Δ**P*_asrC_* strains.**

Expression of *asrC* was determined by qRT-PCR. RNA was extracted from WT and ΔP*_asrC_* strains grown in LB to OD_600_ 0.8. Levels of 5S rRNA were the internal reference. ** *P* < 0.01 compared with WT control group.


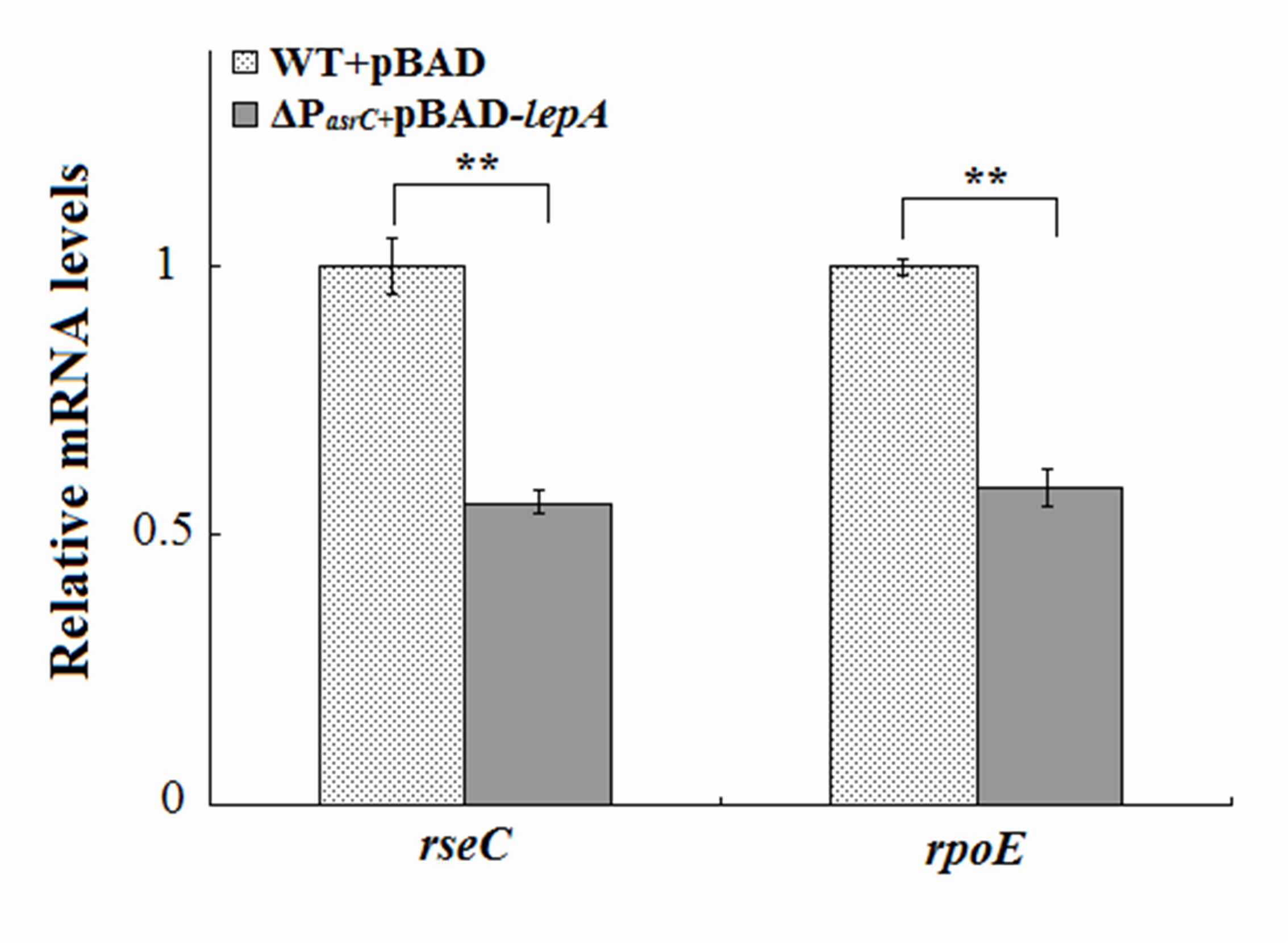


**Figure S2.** **Expressions of *rseC* and *rpoE* in *asrC* mutant strains.**

Expressions of *rseC* and *rpoE* were determined by qRT-PCR. RNA was extracted from WT+pBAD strain and ΔP*_asrC_* *lepA* complementary strain (ΔP*_asrC_*+pBAD-*lepA*) grown in LB to OD_600_ 0.8. Levels of 5S rRNA were the internal reference. ** *P* < 0.01 compared with WT+pBAD control group.
